# Investigating Data Cleaning Methods to Improve Performance of Brain–Computer Interfaces Based on Stereo-Electroencephalography

**DOI:** 10.3389/fnins.2021.725384

**Published:** 2021-10-06

**Authors:** Shengjie Liu, Guangye Li, Shize Jiang, Xiaolong Wu, Jie Hu, Dingguo Zhang, Liang Chen

**Affiliations:** ^1^State Key Laboratory of Mechanical Systems and Vibrations, Institute of Robotics, Shanghai Jiao Tong University, Shanghai, China; ^2^Department of Neurosurgery of Huashan Hospital, Fudan University, Shanghai, China; ^3^Department of Electronic and Electrical Engineering, University of Bath, Bath, United Kingdom

**Keywords:** brain–computer interface, stereo-electroencephalography, data cleaning, re-referencing method, gesture decoding

## Abstract

Stereo-electroencephalography (SEEG) utilizes localized and penetrating depth electrodes to directly measure electrophysiological brain activity. The implanted electrodes generally provide a sparse sampling of multiple brain regions, including both cortical and subcortical structures, making the SEEG neural recordings a potential source for the brain–computer interface (BCI) purpose in recent years. For SEEG signals, data cleaning is an essential preprocessing step in removing excessive noises for further analysis. However, little is known about what kinds of effect that different data cleaning methods may exert on BCI decoding performance and, moreover, what are the reasons causing the differentiated effects. To address these questions, we adopted five different data cleaning methods, including common average reference, gray–white matter reference, electrode shaft reference, bipolar reference, and Laplacian reference, to process the SEEG data and evaluated the effect of these methods on improving BCI decoding performance. Additionally, we also comparatively investigated the changes of SEEG signals induced by these different methods from multiple-domain (e.g., spatial, spectral, and temporal domain). The results showed that data cleaning methods could improve the accuracy of gesture decoding, where the Laplacian reference produced the best performance. Further analysis revealed that the superiority of the data cleaning method with excellent performance might be attributed to the increased distinguishability in the low-frequency band. The findings of this work highlighted the importance of applying proper data clean methods for SEEG signals and proposed the application of Laplacian reference for SEEG-based BCI.

## Introduction

Brain–computer interface (BCI) has been a promising solution toward bridging the gap between disabled people and the external environment (Collinger et al., 2013). Compared with commonly used electroencephalography (EEG), intracranial electroencephalography (iEEG), such as electrocorticography (ECoG), implants grid or strip electrodes directly on the cortical surface and has the characteristics of higher spatial resolution, higher temporal resolution, and higher signal amplitude in the recorded neural signals (Freeman et al., 2000; Ball et al., 2009; Slutzky et al., 2010; Sheehan et al., 2018). In the past 15 years, ECoG-based BCIs have been paid extensive attention (Lebedev and Nicolelis, 2006; Schalk and Leuthardt, 2012; Volkova et al., 2019). Significant achievements have been made for ECoG-based motor BCIs in fine hand movement decoding, such as individual finger classification or hand kinematics regression (Acharya et al., 2010; Flint et al., 2014; Nakanishi et al., 2014; Hotson et al., 2016). Other ECoG studies decoded commonly used functional gestures directly and attained good decoding performance (Pistohl et al., 2012; Chestek et al., 2013; Spueler et al., 2014; Bleichner et al., 2016; Branco et al., 2017). Note that ECoG provides wide coverage over a large area of cortical regions, whereas another increasingly used iEEG recording technique in recent years, stereo-electroencephalography (SEEG), implant multiple depth electrodes into the brain and thus capture neural activities from both cortical and subcortical regions (Parvizi and Kastner, 2018; Herff et al., 2020). Therefore, SEEG provides the unique opportunity to make use of the neural information that cannot be accessed by other invasive techniques. A growing number of studies aiming at testing the possibilities and performance of building a motor BCI using SEEG signals have been reported (Vadera et al., 2013; Murphy et al., 2016; Fischer et al., 2017; Li et al., 2017; Wang et al., 2020).

For SEEG-based BCI, better decoding performance demands a higher quality of acquired SEEG signals, where data cleaning is essential to clean human electrophysiological recordings with nuisance noises. In literature, besides fundamental methods that exclude certain well-defined noise sources (e.g., 50/60 Hz line noise), there is no widely adopted standard for cleaning noise from iEEG data. Both manual and automated approaches are investigated to clean residual noises from the intracranial neural recordings. Specifically, manual methods (e.g., channels or epochs elimination) are used to remove noisy channels or epochs through manual identification (Nolan et al., 2010; Rangarajan et al., 2014; Fonken et al., 2016; Vass et al., 2016; Indira et al., 2017), whereas automated methods, such as common average referencing (CAR) (Schalk et al., 2007), closest white matter reference (CWM) (Arnulfo et al., 2015), bipolar reference (Kobayashi et al., 2009; Vidal et al., 2012; Burke et al., 2014; Greenberg et al., 2015; Sheehan et al., 2018), and Laplacian reference (Mercier et al., 2016; Li et al., 2018), are routinely used to improving signal quality in a global level. It is worth noting that investigators concentrate more attention on the signal itself, but few people assess the performance in BCI applications for these methods. A recent ECoG study has demonstrated that automated methods can produce substantial influence in improving brain state classification compared with manual methods (Meisler et al., 2019). However, for SEEG-based BCI, whether the automated methods have a contribution consistent with the expectation of investigators remains an open question. Accordingly, this paper aims to explore the effects of different automated data cleaning methods for SEEG recordings through practical assessment of gesture decoding performance and, subsequently, propose the optimal one.

Besides, we also comparatively investigated the differences between cleaned SEEG signals using the optimal method and other automated methods by conducting a multiple domains analysis. Finally, the reasons causing the differentiated effects were explored.

## Materials and Methods

### Subjects

Eight subjects with intractable epilepsy participated in this study. They had SEEG electrodes implanted for pre-surgical assessment of their seizure focus. The recording information of each subject is shown in Table 1. The subjects performed the task using hand contralateral to the hemisphere with the majority of electrodes implantation. The Ethics Committee of Huashan Hospital approved the study (Shanghai, China, approval ID: KY2019518), and informed consent for the study was obtained from all subjects.

**TABLE 1:** Experimental information of subjects that participated in study.

ID	RS	SR (Hz)	NES	NC
1	Left	1,000	10	121
2	Left	1,000	15	180
3	Right	1,000	7	60
4	Left	2,000	16	208
5	Left	2,000	7	102
6	Left	2,000	10	144
7	Left	2,000	8	110
8	Right	2,000	15	190

*RS, recording hemisphere; SR, sampling rate; NES, number of electrode shafts; NC: number of contacts.*

### Data Recording

SEEG signals were recorded using a clinical recording system (EEG-1200C, Nihon Kohden, Irvine, CA) system with 1,000- or 2,000-Hz sampling rates (Table 1). The original SEEG signals collected by the system were referenced to the average of two adjacent white matter contacts locating remotely from the suspected seizure focus and gray matter. To monitor the actual movement onset time during the experiment, two surface electromyographic (EMG) electrodes were used to record EMG signals from extensor carpi radialis muscle with the same sampling rate and same recording device as the SEEG signals simultaneously.

### Experimental Protocol

**FIGURE 1:**
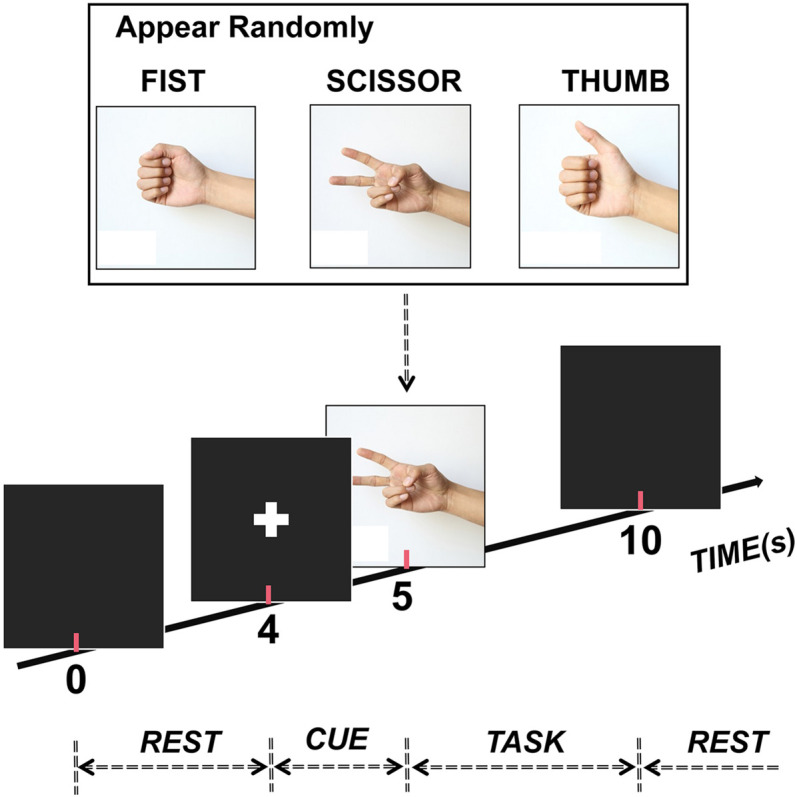
Experimental paradigm. Each trial lasted 10 s (4-s rest, 1-s cue, and 5-s task). Also, one of three hand gestures (scissor, fist, and thumb) randomly appeared to inform participants to execute the corresponding movement during the task stage.

The subjects participated in a visually cued hand movement task during the experiment (Figure 1). In detail, at the beginning of each trial, the subject faced an LED screen and took a rest for 4 s first without any hand movement before a cue (white cross, 1 s) appeared to inform them of the upcoming movement. After this, a picture illustrating one of three gestures (scissor, fist, and thumb) appeared on the screen randomly. Then, the subject executed the corresponding movement repeatedly for 5 s as soon as the appearance of a picture. Twenty trials were performed for each gesture, making the data from a total of 60 trials were collected for each subject.

### Electrode Localization

**FIGURE 2:**
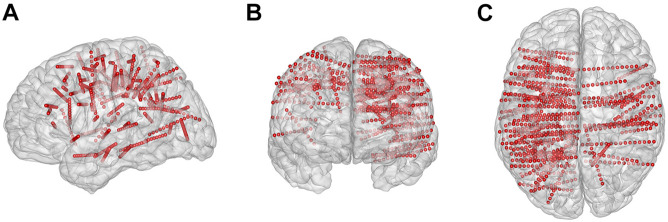
Electrode locations projected on the three-dimensional standard Montreal Neurological Institute brain model. **(A–C)** Brain model and implanted contacts (small red dots) in the sagittal, coronal, and transverse view, respectively.

The subjects had a total of 88 electrode shafts (rounded mean ± std: 11 ± 4 per subject) and 1,115 contacts (rounded mean ± std: 139 ± 50 per subject) implanted (Figure 2). Each electrode contains 8–16 contacts, and each contact was 2.0 mm long with a 0.8-mm diameter and a 3.5-mm center-to-center spacing distance. We identified the location of all contacts in each individual brain model using pre-surgical magnetic resonance imaging, post-surgical computed tomographic images, Freesurfer software^1^, and the iEEGview toolbox (Li et al., 2020).

### Automated Data Cleaning Methods

The main goal of this study was to evaluate the potential influence of different data cleaning methods on decoding performance for SEEG-based BCI. To do this, five previously used automated methods (Li et al., 2018), including common average reference (CAR), gray–white matter reference (GWR), electrode shaft reference (ESR), bipolar reference, and Laplacian reference, were adopted for the evaluation.

For the CAR method, re-referencing was achieved by first creating an average of all channels and then subtracting the average signal from each channel (Kubanek et al., 2009; Miller et al., 2012). Slightly differently, for GWR, we re-referenced each channel located in the gray or white matter to the corresponding average of all gray and white matter channels separately. To do the ESR, we re-referenced each channel to the average signal of all channels located on the same shaft. For bipolar reference (Kobayashi et al., 2009; Vidal et al., 2012; Shirhatti et al., 2016), each channel was re-referenced to its adjacent channel on the same electrode shaft. Finally, to implement the Laplacian reference (He et al., 2008), each contact was re-referenced by the mean value of two adjacent contacts along the electrode shaft.

Besides, to answer the question of whether automated data cleaning can benefit decoding performance, we treated the signal without any re-referencing process (termed as a raw signal in this work) as a benchmark and conducted the same following analysis for these signals as well.

### Data Preprocessing

**FIGURE 3:**
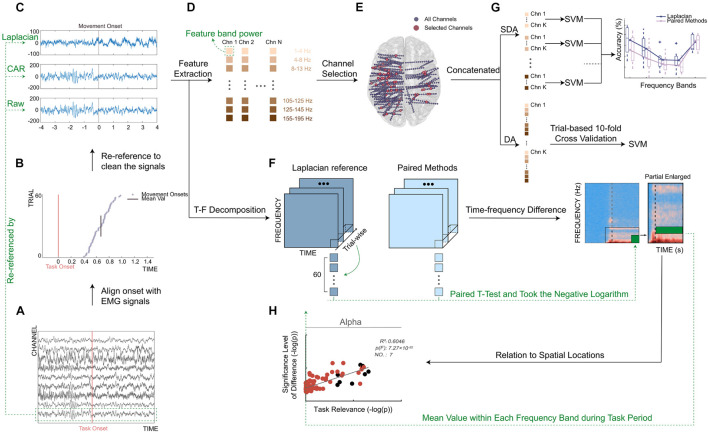
Framework of this work. **(A)** Single-trial filtered signals of selected channels. Redline represented task onset (appearance of movement cue, Figure 1). **(B)** Distribution of movement onsets across trials. Redline represented task onset, and purple asterisks represented detected movement onsets, and blackline represented mean value of movement onsets across trials. **(C)** A typical example of signals re-referenced by Laplacian reference, CAR, and Raw. Signals from one trial of a typical channel were shown. Blackline represented mean value of movement onsets across trials. **(D)** Feature extraction. Colored squares represented different frequency band powers. **(E)** Distribution of implanted channel (small blue dots) and selected channel (big red dots) from all subjects. All electrodes were projected on a standard Montreal Neurological Institute brain model in a transverse view. **(F)** Illustration of a calculation process for time–frequency difference (TFD). **(G)** Illustration of single-frequency band-based decoding accuracy (SDA). Single sub-band power of selected channels was taken separately to build the feature vector. **(H)** Relation to spatial locations. Take mean value of TFD within each frequency band during task period to calculate significance level of spectral difference.

For the collected original SEEG signals, we implemented the following preprocessing procedure. First, we resampled the SEEG signals to 1,000 Hz using the function (*resample*) in Matlab (MathWorks, Natick, MA, United States) to reduce computational cost and facilitate further computation across subjects. Then, we calculated a measure of line noise to remove channels with excessive line noise. Specifically, a second-order IIR peak filter (*iirpeak*) at 50 Hz was first applied to retain the 50-Hz frequency component. Then, we concatenated the filtered signals from all channels of each subject to calculate a cutoff threshold for noisy channels. Also, the threshold was defined as the summation of the median of the concatenation output and 10 times of its mean absolute deviation. The channels whose line noise was higher than the cutoff threshold were identified as bad channels and eliminated from further analysis. Then, a 50-Hz comb notch filter was applied to remove the possible line noise and its harmonics. After that, a 0.5–400 Hz band-pass filter (fourth-order Butterworth) was used to filter the resampled signal (Figure 3A). At the last step, the respective data cleaning methods described earlier (*Automated data cleaning methods*) were applied to clean the filtered signals (Figure 3C).

Additionally, the obtained EMG signals were processed as well. In detail, we band-pass filtered (55–145 Hz, sixth-order Butterworth filter) the two EMG channels and subtracted the results from each other. Notably, such an adopted filtering setting can ensure the accurate detection of movement onsets’ use of high-frequency information of EMG signals. Then, for each trial, we detected the point where the absolute amplitude of EMG signals exceeded first time an adaptive threshold using the envelope of the processed EMG activity (Sedghamiz, 2018). Also, we detected the point where absolute EMG activity first time exceeded 1.5 times the average absolute value of EMG activity in the task stage. Finally, the earlier of the two points was treated as movement onset to ensure a robust EMG activity onset detection (Wang et al., 2020).

Finally, the SEEG signals were aligned to the movement onset (mean ± std: 0.72 ± 0.38 s after the onset of gesture picture) for each trial and each subject (Figure 3B). To be concise, the movement onset was defined as time 0. The baseline period and task period were defined as the [−4, −2] s and [0, 4] s around the movement onset, respectively. In each trial, the signals within [−4, 4] s around the movement onset were used in the following decoding analysis.

### Performance Index–Decoding Accuracy

To quantify the decoding performance under different data cleaning methods, gesture decoding accuracy (DA) was computed. To do this, we conducted a gesture decoding procedure for each method and each subject separately. In detail, for each channel, we first segmented the cleaned signals from the task period using a time window at a length of 500 ms (overlapping by 250 ms) and then transformed the signals of each time bin into frequency domain with an autoregressive model of order 40 (Li et al., 2017). The spectral power in the sub-bands (e.g., 1–4, 4–8, 8–13, 13–30, 60–75, 75–95, 105–125, 125–145, and 155–195 Hz) were extracted by averaging transformed data within each interval (Figure 3D). After that, we normalized (i.e., *Z*-score) the obtained spectral power to the average power of the baseline period of all trials. In the last step of feature extraction, the normalized power from all frequency bands was concatenated together to build the feature vector for each channel.

Considering that SEEG electrodes were widely distributed inside the brain, channel selection was extremely necessary for the decoding purpose (Figure 3E). In this step, we used the forward search optimization algorithm to select the optimal channel set for each subject (see also Supplementary Figure 1; Kohavi and John, 1997; Lotte et al., 2018). More specifically, starting from the empty optimal channel set, we successionally selected one from all remaining channels, which produced the highest accuracy with the selected channels and incorporated it into the optimal channel set. To avoid the possible local extreme point during searching, the iteration was stopped when the accuracies reached a peak and did not increase after three more channels were added. Then, for each sample (i.e., time window), the feature vectors from all channels in the optimal channel set were concatenated together to construct the final feature vector for the classifier. Moreover, to achieve a robust classification, each element of the obtained final feature vector was renormalized across trials to maximally eliminate the difference of absolute amplitude among channels (Wang et al., 2020).

Finally, a Support Vector Machine classifier with a linear kernel was used for the classification of multiple hand gestures. Moreover, considering the data overlapping during the feature extraction, to avoid data leakage and make a fair evaluation for this process, we adopted trial-based 10-fold cross-validation. In brief, in each cross-validation, we first randomly selected ninefold (*n* = 54) of all trials (*n* = 60) as training set and the remaining onefold as testing set. For each subject and each method, the decoding accuracy was computed as the mean decoding value of all 10-fold cross-validation.

### Comparison Indices

To further interpret the reasons causing the differentiated performances between the best-performing method and other data cleaning methods, three comparison indices were proposed and computed: (1) TFD, (2) SDA, and (3) relation to spatial locations.

#### Time–Frequency Difference

TFD was proposed in this work to quantify the spectral and temporal difference between different data cleaning methods. To compute the TFD, for each selected channel of each subject under each automated data cleaning method, we used the fast Fourier transform together with a Hanning window (*timefreq* function from EEGlab toolbox; Delorme and Makeig, 2004) for the purpose of time–frequency decomposition of trials (Band limits: 0–195 Hz) and stacked the decomposition of all trials to generate an array with three dimensions along time (*t*), frequency (*f*), and trials (*i*) [termed as *TFT*(*t,f,i*) here]. Then, we calculated trial-wise paired *t*-test of the *TFT(t,f,i)* between paired data cleaning methods (e.g., the optimal method and the other ones) and took the negative logarithm of the *p*-value for each element [e.g., −log(*p*(*t, f*))] to obtain the TFD of a single channel. Notably, given the multitude of selected channels for each subject and the fact of electrical field spread (leading to source leakage and volume conduction), Bonferroni correction was adopted for the *p*-values to correct the family-wise error. Finally, the distinguish index TFD (*t, f*) was calculated as the average across all selected channels of all subjects, where *-log(0.05)* was taken as the significance level (Figure 3F). Considering that the optimal channel set of different methods might be different, the selected channel set here was the union of the optimal channel sets of the two paired methods.

#### Single-Frequency Band-Based Decoding Accuracy

After evaluating the changes of data cleaning methods exerted to the signal itself, we further investigated how the induced changes affected the decoding performance. For this purpose, we calculated the SDA for different data cleaning methods. In detail, similar to the calculation of DA (*Performance index–decoding accuracy*), we separately took the single sub-band power (e.g., 1–4, 4–8, 8–13, 13–30, and 60–195 Hz) of the selected channels used in DA calculation to build the final feature vector (Figure 3G). Among these sub-bands, considering that high gamma band (60–195 Hz) had a comparatively larger frequency span, this band was segmented into multiple bins (e.g., 60–75, 75–95, 105–125, 125–145, and 155–195 Hz) to fully extract the effective neural information during the feature extraction. After this, a Support Vector Machine classifier (same as *Performance index–decoding accuracy*) was adopted to calculate the SDA for each subject and each data cleaning method.

#### Relation to Spatial Locations

To further answer the question of whether the differences induced between data cleaning methods have a relationship with spatial locations, we calculated two indices and measured the correlation between these two values. The first one is the significance level of spectral difference between the optimal data cleaning method and the other ones, where this value quantifies how much difference the two data cleaning methods exerting on the SEEG signal itself. The second index is the task relevance, and this value quantifies how much the SEEG channels correlate with the task. These two indices were computed for each channel and each subject. Moreover, to more precisely evaluate the potential relationship, the analysis was conducted using multiple frequency bands (e.g., 1–4, 4–8, 8–13, 13–30, and 60–195 Hz) separately.

To be more specific, for the calculation of the significance level of spectral difference, we took the mean value of the TFD (*t, f*) within each frequency band during the task period (*Time–frequency difference*) between the optimal method and the other methods (Figure 3H, see also Supplementary Figure 2). To calculate task relevance, we took the correlation between the single-frequency band power of each channel and the task. In detail, separately for each frequency band, we first applied a band-pass filter to signals processed by the optimal data cleaning method at this frequency band using a sixth-order Butterworth filter. Then, we extracted frequency band power by computing the squared absolute value of the Hilbert transform and obtained the mean frequency band power of the baseline period and task period for each trial separately. Second, we correlated (Spearman’s correlation coefficient) these power values of all trials with the corresponding task/baseline labels (e.g., 0/1) to get an observation of the *r* value. Third, we performed a permutation test in which we randomly shuffled the task/baseline labels and calculated the corresponding random *r* value (Schalk et al., 2007). The permutation test was repeated 1,000 times and generated a Gaussian distribution of 1,000 surrogate *r* values. The channel was considered statistically task-related if the computed channel *r* belonged to the 95th percentile of the Gaussian distribution (*p* < 0.05 after Bonferroni correction). Finally, task relevance was taken as the negative logarithm of the *p*-value [i.e., *-log(p)*].

After this, to determine whether there was a relationship between the significance level of spectral difference and the task relevance of each channel, we performed a first-order linear fitting using the function (*polyfit*) in Matlab. Moreover, *F*-test was used for regression analysis to assess the significance of the fitting equation (Shen and Faraway, 2004; Demidenko et al., 2012), where the function (*stats.f.sf*) from SciPy toolbox (Jones et al., 2001) was adopted to calculate the *p*-value of the *F*-test. Finally, the coefficient of determination (i.e., *R*-square) was calculated to evaluate the fitness of the line fitting.

## Results

### Decoding Accuracy of Different Data Cleaning Methods

**FIGURE 4:**
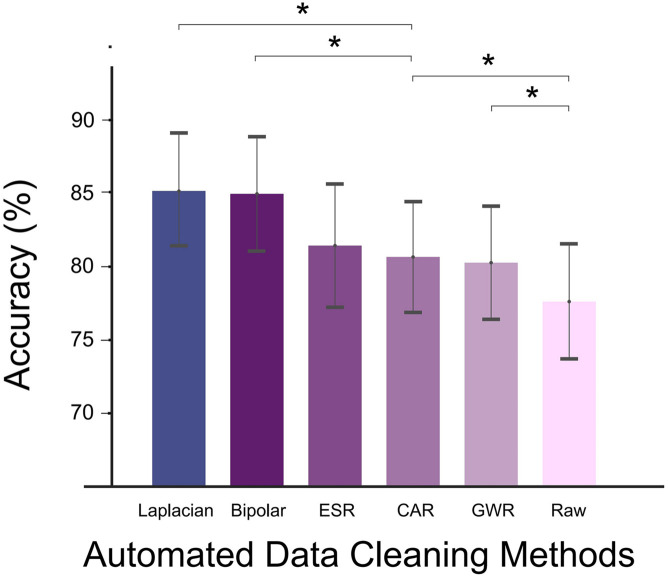
Decoding accuracy (DA) of different automated data cleaning methods (Laplacian reference, bipolar reference, ESR, CAR, GWR, and Raw). Asterisks denoted statistical significance level of accuracy value between groups (*p < *0.05*, paired *t*-tests). Bars and error bars represented mean (across all subjects) accuracy and standard error, respectively. Bars arranged in descending order according to average accuracy value.

The DA calculated under different data cleaning methods is shown in Figure 4. Compared with the signals without re-referencing (Raw in Figure 4), all data cleaning methods produced significantly higher DA values (*p* < *0.05*, paired *t*-test), demonstrating that the adoption of proper data cleaning methods for SEEG signals could effectively enhance the decoding performance.

Among all the applied data cleaning methods, Laplacian reference produced the highest DA in average, reaching 85.2 ± 3.8% (mean ± s.e.). Bipolar and ESR ranked the second and third places; their DAs were lower than the Laplacian method by 0.2 and 3.7%, respectively. For CAR and GWR, the enhancing effects were weakest, and their DAs were significantly lower than Laplacian (*p* < *0.05*, paired *t*-test, Figure 4).

### Time–Frequency Difference Between Paired Data Cleaning Methods

**FIGURE 5:**
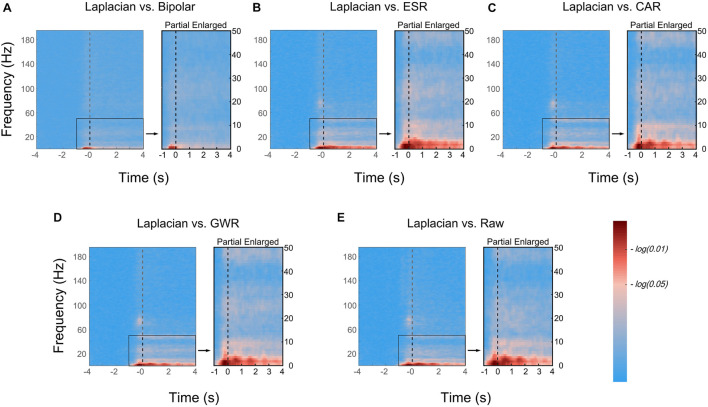
Time–frequency difference (TFD) between paired data cleaning methods. **(A)** Laplacian reference versus bipolar reference; **(B)** Laplacian reference *versus* ESR; **(C)** Laplacian reference *versus* CAR; **(D)** Laplacian reference *versus* GWR; **(E)** Laplacian reference *versus* Raw. Difference in low-frequency region was partially enlarged for visualization purpose (Time, [−1, 4] s; Frequency, [0, 50] Hz). Colors represented TFD value between paired methods. Black dotted line represented movement onset, and [−4, 4] s around movement onset were presented.

We have shown that the Laplacian method produced the best decoding performance in the previous section (*Decoding accuracy of different data cleaning methods*). To further investigate which factors may contribute to this phenomenon, we calculated the TFD between the Laplacian reference and other automated data cleaning methods (Figure 5). As could be seen from the figure, the significant difference between the Laplacian reference and ESR, CAR, GWR, and Raw was mainly reflected in the low-frequency band (LFB, e.g., delta, theta, and alpha bands), where the TFD (*t, f*) was higher than the significance level [*−log(0.05)*], especially in the delta band (1–4 Hz) that was showing a more significant difference [TFD (*t, f*) > *−log(0.01)*]. In the temporal domain, the significant difference was mainly distributed in the [−1, 4] s around the movement onset. Comparing Laplacian reference with bipolar reference, the significant difference occurred in delta band during the [−0.5, 0] s around the movement onset (after the gesture picture appeared and before moving), and there was no significant difference during the task period. For all paired methods, no significant difference was seen during the baseline period.

### Single-Frequency Band Based Decoding Accuracy for Different Data Cleaning Methods

**FIGURE 6:**
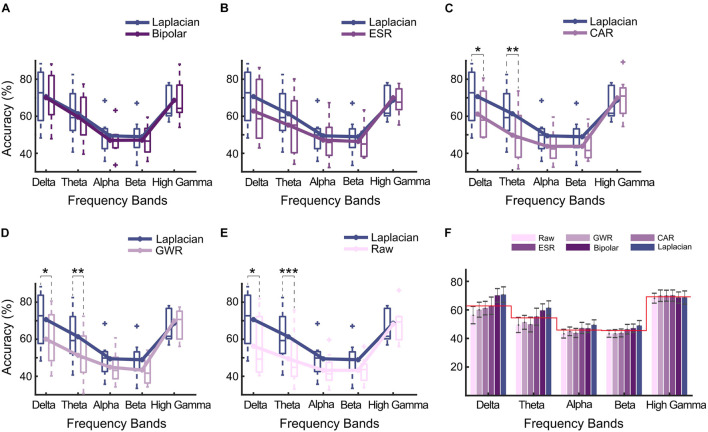
Single-frequency band-based decoding accuracy (SDA) of data cleaning methods for multiple sub-bands. **(A)** Laplacian reference *versus* bipolar reference; **(B)** Laplacian reference *versus* ESR; **(C)** Laplacian reference *versus* CAR; **(D)** Laplacian reference *versus* GWR; **(E)** Laplacian reference *versus* Raw. Sub-bands included delta band (1–4 Hz), theta band (4–8 Hz), alpha band (8–13 Hz), beta band (13–30 Hz), and high gamma band (60–195 Hz). Asterisks denoted statistical significance level of SDAs between paired methods (***p < *0.001*; **p < *0.01*; *p < *0.05*, paired *t*-test). Box and line represented boxplot and mean accuracy across all subjects. **(F)** Comparison of SDAs of different frequency bands. Bars and error bars represented mean accuracy and standard error calculated across all subjects, respectively. Redline represented average SDAs across all methods for different bands, respectively.

To further explore whether it is the spectral difference that caused the differentiated decoding performance between paired methods, we calculated the SDAs of different methods (*Single-frequency band-based decoding accuracy*). The results are presented in Figure 6. Overall, the Laplacian method achieved the highest SDAs. More specifically, compared with CAR, GWR, and Raw, the SDAs of Laplacian reference were significantly higher in delta (*p* < *0.05*, Bonferroni corrected, paired *t*-test, Figures 6C–E) and theta (*p* < *0.01*, Bonferroni corrected, Figures 6C–E). Compared with ESR, the Laplacian method produced higher SDAs on average but without significance. For the comparison between bipolar and Laplacian references, these two methods produced very similar results. No significant difference between the SDAs of Laplacian reference and other methods in a high gamma band was seen. Notably, the statistical properties of SDAs in LFB (e.g., delta and theta bands) and high gamma band (60–195 Hz) were highly consistent with those observed in TFD (Figure 5, *Time–frequency difference between paired data cleaning methods*).

On average, a high gamma band produced higher classification accuracy (69.2%, Figure 6F). On the contrary, the average SDAs across all methods in LFB were 5.7% (delta), 14.8% (theta), 23.3% (alpha), and 23.7% (beta) lower than that of high gamma band. Interestingly, among all automated methods, Laplacian reference and bipolar reference presented higher accuracy in delta band than the high gamma band, where the SDA of these two methods were 1.5 and 2.0% higher than that of high gamma band, respectively (Figure 6F).

### Relationship Between Spatial Locations and Significance Level of Spectral Difference for Paired Methods

**FIGURE 7:**
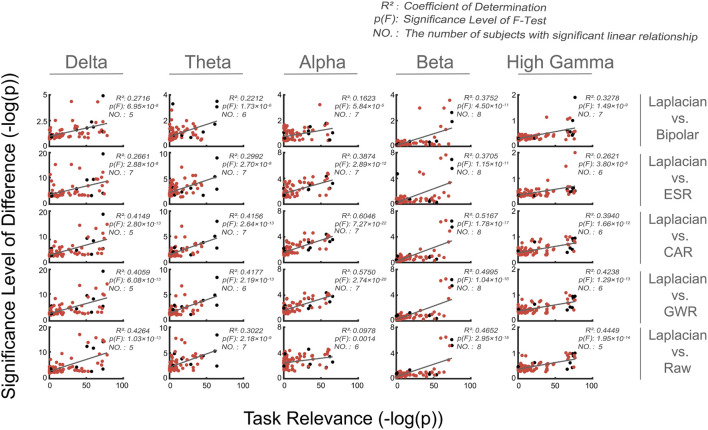
Relationship between task relevance and significance level of spectral difference for all channels from a typical subject (Subject 5). Frequency bands in delta, theta, alpha, beta, and high gamma band were analyzed, and five paired methods (Laplacian reference *versus* bipolar reference, ESR, CAR, GWR, and Raw, respectively) were presented. Each red dot represented one channel from the subject, and black dots represented the selected channels used for decoding under Laplacian reference (*Performance index–decoding accuracy*). In the subgraph, the x-axis represented the task relevance of channels, the y-axis represented the significance level of spectral difference of channels, and the straight gray line was the first order linear fitting of task relevance and significance level of difference. Three indices were presented (*R*-square, *p*-value of *F*-Test, and the number of subjects with significant linear relationship).

In the analysis mentioned earlier, we took the channels as a whole and calculated them in the mean sense and, thus, largely ignored the spatial (i.e., channel) information. In this section, we investigated the question of whether the difference between paired data cleaning methods would be more significantly presented on the channels that are more related to the task (*Relation to spatial locations*). Figure 7 shows the relationship between task relevance of all channels and the corresponding significance level of differences for all paired data cleaning methods (Laplacian versus others) in multiple frequency bands from a typical subject (Subject 5). As could be seen from Figure 7, there existed a significant linear relationship (*p* < *0.05*, *F*-test) between the two indices for almost all subjects and all frequency bands (see also Supplementary Figure 3). In addition, for Laplacian reference, 85% (i.e., 74/87) of the selected channels across all subjects were task-related in a certain frequency band. Altogether, the results indicated that the Laplacian method could produce more prominent improvements to the signals of the most informative channels compared to other methods.

## Discussion

In this study, we provided the first systematical evaluation of several automated data cleaning methods routinely used in SEEG studies, with the goal of verifying the effects of these methods and proposing the optimal method for SEEG-based BCI.

The results in this study showed that applying automated methods to a clean SEEG signal was able to improve the decoding accuracy. Among the listed methods, the Laplacian reference had the best performance. The current finding was in accordance with the previous SEEG study (Li et al., 2018), where Laplacian reference had also been demonstrated to be optimal in improving global signal quality metrics (e.g., the correlation of signals across channels). One step further, this work answered the subsequent question that the optimal SEEG data cleaning method is targeted for BCI purposes (Figures 5–7). The agreement reached in these two evaluation studies altogether suggested the application of Laplacian reference for further SEEG research.

To interpret the reasons causing the differentiated performances across data cleaning methods, we comparatively investigated the multiple-domain (e.g., spatial, spectral, and temporal domain) changes of SEEG signals. The results in Figure 5 shows that the Laplacian reference had a significant spectral difference in the LFB (e.g., delta, theta, and alpha bands) compared with the other automated methods (such as CAR and GWR, see also Supplementary Figure 2). Furthermore, we decoded the hand gestures based on a single-frequency band and found that the decoding performance of the Laplacian reference in LFB (e.g., delta and theta bands) significantly outperformed CAR and GWR (Figures 6A–E). Current results are also in agreement with the previous study, where Laplacian reference has been shown to be able to enhance the relationship with the task for LFB (e.g., alpha) power activity (Li et al., 2018).

Besides, in the frequency band, the power of the high-frequency band has been demonstrated to be correlated with the population-level cortical activity associated with different motor, sensory, or cognitive tasks (Gaona et al., 2011; Ray and Maunsell, 2011; Miller et al., 2014; Potes et al., 2014; Pesters et al., 2016; Branco et al., 2017) and, therefore, generally yield a higher classification accuracy than the low-frequency range (e.g., 8–32 Hz) in multiple tasks decoding (Ojemann, 2007; Kai et al., 2010; Gruenwald et al., 2017). The consistent results were found in this work (Figure 6F). Notably, several pieces of evidence tended to support the notion that the delta band also has an important effect on motion execution. Specifically, Graimann et al. (2004) showed a clear correlation between the normalized band power changes in the delta range with movement execution. Aleksandra found that the delta band carried significant motor-related information in classifying real wrist movements (Vuckovic and Sepulveda, 2008). In this work, the delta band had been found to be able to achieve the highest accuracy among the LFB of all automated methods, which highlighted the importance of the delta band in motion execution decoding (Schalk et al., 2007; Pistohl et al., 2012). Moreover, the delta band even achieved similar accuracy with the high-frequency band under the Laplacian or bipolar method in this work. One explanation for this result may be that the repetitive hand movement in our experiment required multiple movement initiation, which was modulated by delta band amplitude (Kobler et al., 2020). However, the reasons behind this result still need further investigation. Overall, the findings of this work highlight the importance of taking the high gamma band and delta band into consideration together for further BCI application.

Although we have presented the optimal SEEG data cleaning method for BCI usage based on the data observations, this work also has some limitations. First, for the comparison between Laplacian and other locally processed automated data cleaning methods (e.g., Bipolar reference and ESR), the Laplacian method held advantages on performance indicators on average but without significance (Figures 5, 6). This may be because of the limited number of subjects. Therefore, further study using a large number of subjects is still necessary for the next step. Second, in this study, we only used a DA to measure the effect of different automated methods on SEEG-based BCI without showing the anatomical information of the used key decoding electrodes. Due to the wide coverage in both cortical and subcortical levels for SEEG recordings, the spatial analysis on different brain regions is also of great importance for BCI research, which will be conducted in future work. Third, as the following study of our previous work (Li et al., 2018), we conduct analysis among the same different data cleaning methods and draw the conclusion within these tested methods. Although we also notice that some other data cleaning methods have been reported for SEEG recordings (Arnulfo et al., 2015; Schaworonkow and Voytek, 2021), further comparison between the Laplacian with these methods will be meaningful and thus worth of exploration in the future.

## Conclusion

This study mainly explored the influence on the decoding performance of five automated data cleaning methods commonly used in SEEG studies. Moreover, we further investigated why the different methods may result in different decoding performances. The result showed that Laplacian reference produced the best enhancing effect on decoding performance, and such phenomenon may be caused by the increased ability on the task information retainment of low-frequency band activities compared with other data cleaning methods. This study provided practical guidance for the data cleaning method to be used for further BCI applications based on SEEG signals.

## Data Availability Statement

The raw data supporting the conclusions of this article will be made available by the authors, without undue reservation.

## Ethics Statement

The studies involving human participants were reviewed and approved by Ethics Committee of Huashan Hospital (Shanghai, China, Approval ID: KY2019518). The patients/participants provided their written informed consent to participate in this study.

## Author Contributions

SL, GL, LC, and DZ: conceptualization. SL, GL, and XW: methodology. SL and GL: software, writing—original draft, and visualization. SL: formal analysis. SL, GL, SJ, DZ, and LC: investigation. SJ: data curation. SJ, XW, and DZ: writing—review and editing. DZ, LC, and JH: supervision. DZ and LC: project administration. DZ, LC, and GL: funding acquisition. All authors read and approved the final version of the manuscript.

## Conflict of Interest

The authors declare that the research was conducted in the absence of any commercial or financial relationships that could be construed as a potential conflict of interest.

## Publisher’s Note

All claims expressed in this article are solely those of the authors and do not necessarily represent those of their affiliated organizations, or those of the publisher, the editors and the reviewers. Any product that may be evaluated in this article, or claim that may be made by its manufacturer, is not guaranteed or endorsed by the publisher.

## Footnotes

1
http://surfer.nmr.mgh.harvard.edu
