# Supplementary material for: Investigating Data Cleaning Methods to Improve Performance of Brain–Computer Interfaces Based on Stereo-Electroencephalography
Source: Front Neurosci. 2021 Oct 6;15:725384. doi: 10.3389/fnins.2021.725384 (PMC8528199; doi:10.3389/fnins.2021.725384)
Supplement: Supplementary file 1 [file Data_Sheet_1.docx]

Supplementary Material

# Supplementary Figure Legends

**Supplementary Figure 1.** The locations of implanted contacts and selected contacts from each subject. The contacts were projected on the three-dimensional standard Montreal Neurological Institute brain model. For each subject, three panels showed the implanted contacts (small blue dots) and the selected contacts (big red dots) in the sagittal, coronal, and transverse view respectively.

**Supplementary Figure 2.** Significance level of spectral difference of all channels between Laplacian reference and CAR. Each subfigure was generated by averaging the TFD values between paired methods in the task period for each channel (i.e., folding the time axis, See Fig.5). Colors represented the -log(*p*) value (Bonferroni corrected) between paired methods. For each subject, the x-axis represented channels, and the y-axis represented frequency.

**Supplementary Figure 3.** Relationship between task relevance and significance level of spectral difference for channels of all subjects. Frequency bands in delta, theta, alpha, beta and high gamma band were analyzed and five paired methods (Laplacian reference versus bipolar reference, ESR, CAR, GWR and Raw respectively) were presented. Each red dot represented one channel from the subject and black dots represented the selected channels of Laplacian reference. In subgraph, x-axis represented the task relevance of channels, and y-axis represented the significance level of spectral difference of channels, and straight grey line was the first order linear fitting of TR and significance level of difference. Asterisks denoted the significance of the fitting equation (***, *p<0.001*; **, *p<0.01*; *, *p<0.05*, F test).
