# Supplementary figures and images for: Investigating Data Cleaning Methods to Improve Performance of Brain–Computer Interfaces Based on Stereo-Electroencephalography
Source: Front Neurosci. 2021 Oct 6;15:725384. doi: 10.3389/fnins.2021.725384 (PMC8528199; doi:10.3389/fnins.2021.725384)

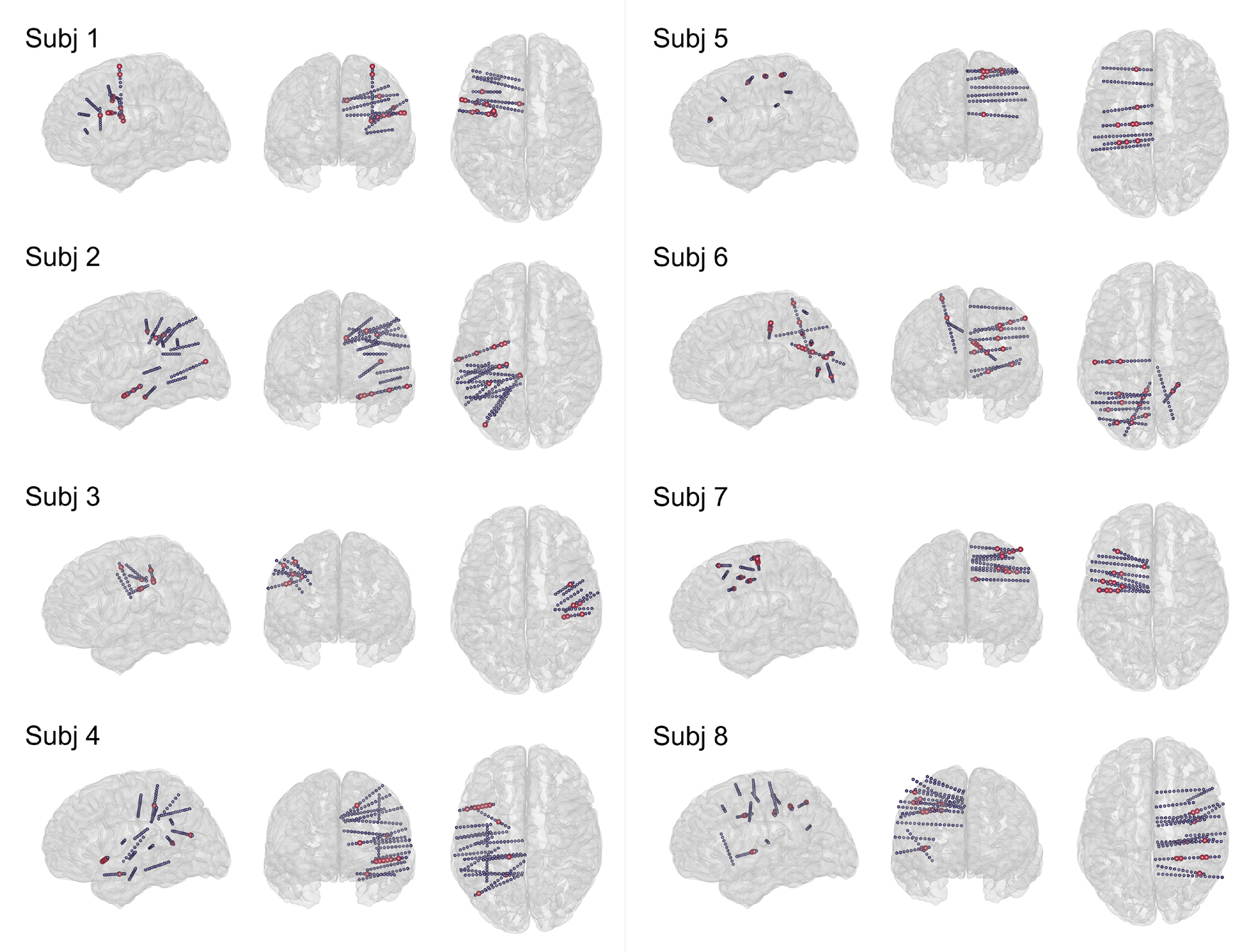

Supplement: Supplementary file 2 [file Image_1.JPEG]

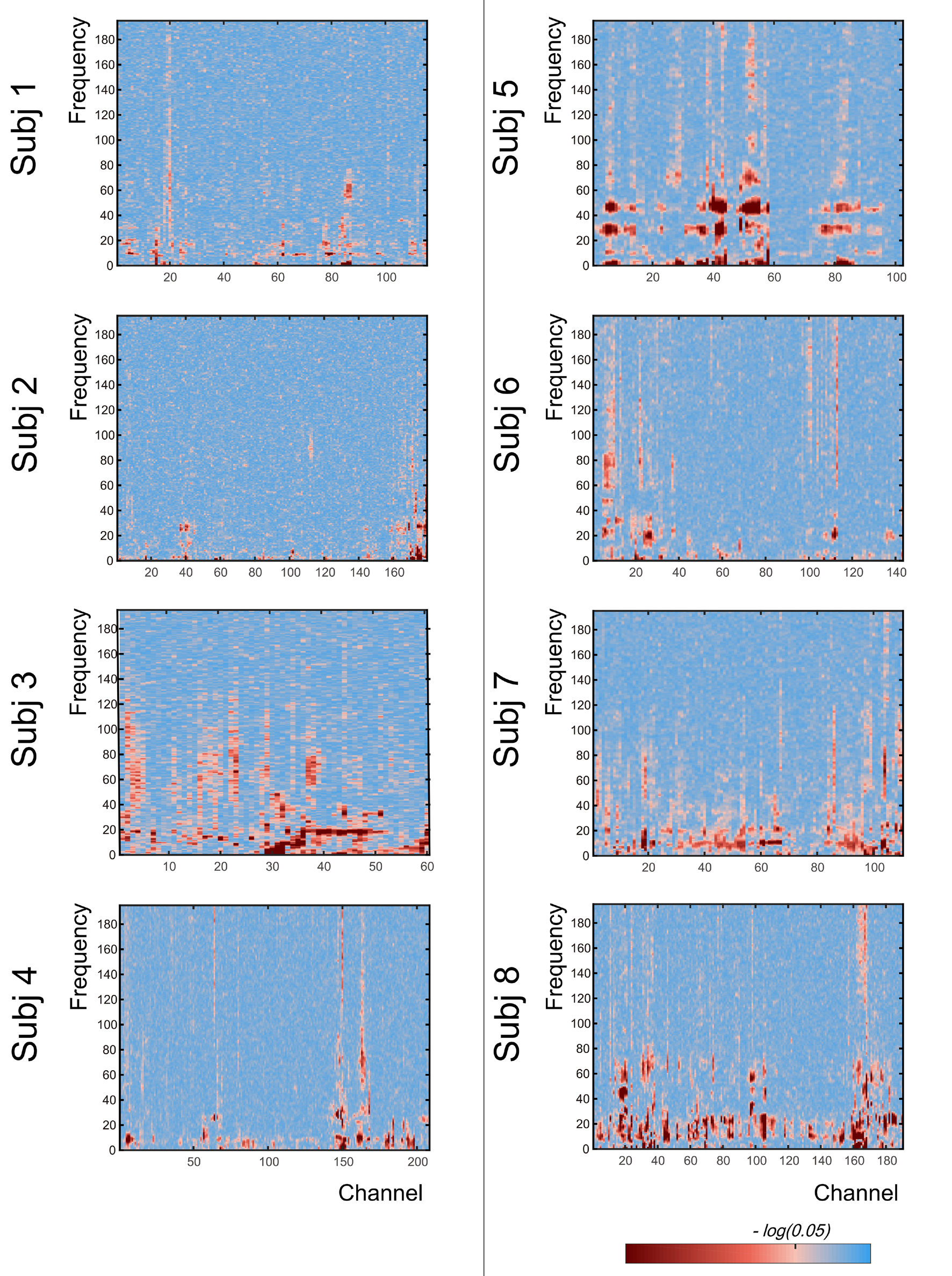

Supplement: Supplementary file 3 [file Image_2.JPEG]

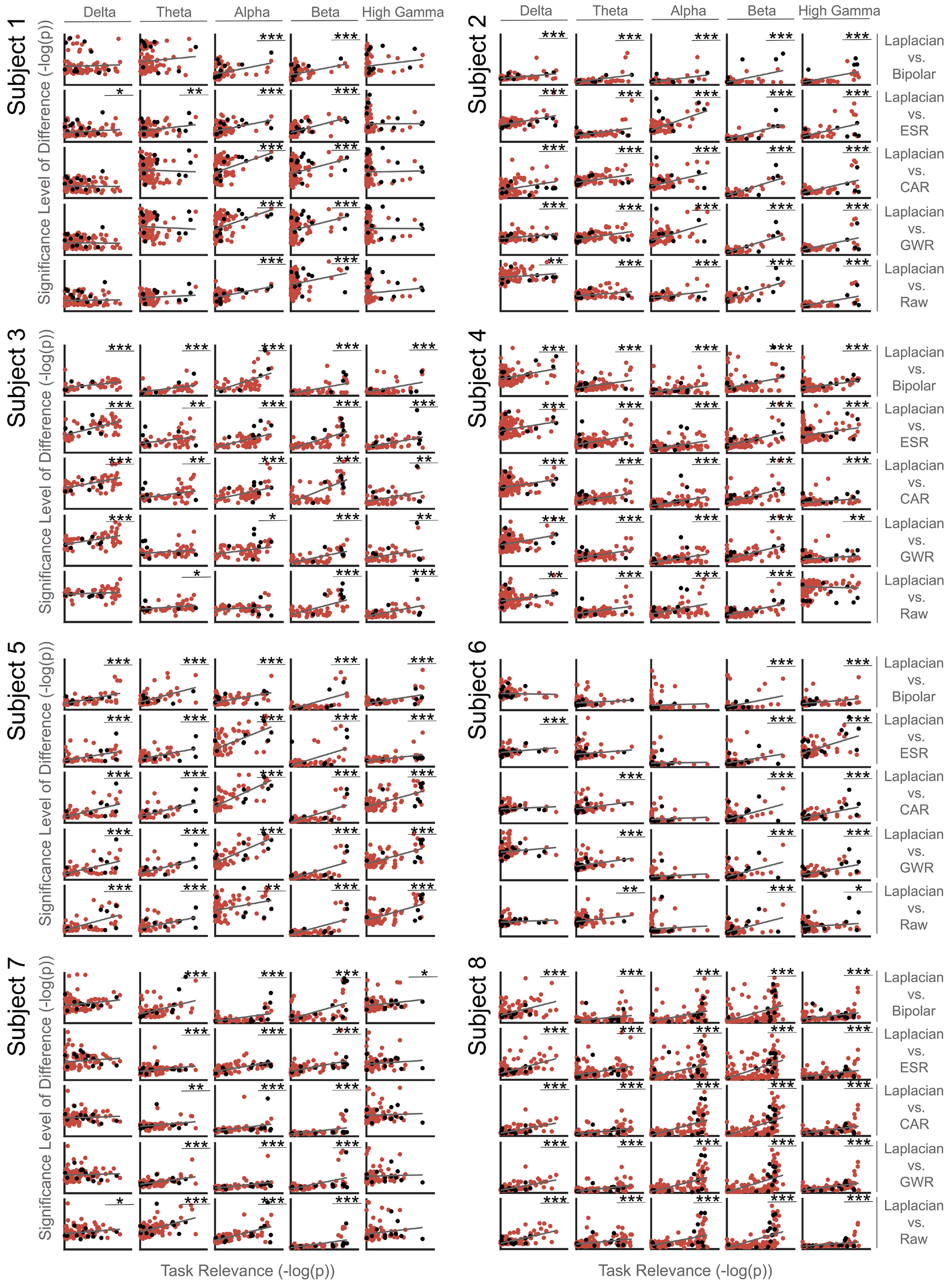

Supplement: Supplementary file 4 [file Image_3.JPEG]
